# An Efficient Biological Codon Recognition Reservoir Computing System Based on Low‐Energy Epitaxial Hf_0.52_Zr_0.48_O_2_ Ferroelectric Memristors

**DOI:** 10.1002/advs.202600021

**Published:** 2026-09-09

**Authors:** Ying Liu, Jikang Xu, Yongqing Jia, Wenxuan Wang, Weifeng Zhang, Biao Yang, Xiaobing Yan

**Affiliations:** ^1^ Key Laboratory of Brain‐like Neuromorphic Devices and Systems of Hebei Province College of Electronic and Information Engineering Hebei University Baoding People's Republic of China

**Keywords:** biological codon recognition reservoir computing system, epitaxial ferroelectric memristors, high endurance, low energy, multilevel storage

## Abstract

The large demand for information processing has stimulated interest in low‐power and fast‐storage hafnium‐based ferroelectric memristors because of their ability to precisely control the state of the resistor by polarization flip‐flop without the need for electroforming. However, there is still a lack of hafnium‐based ferroelectric memristor with both high stability and ultra‐low operating energy consumption, which are the basic conditions for efficient neural network computation with high recognition rates. This article introduces a high‐quality epitaxially grown Pd/Hf_0.52_Zr_0.48_O_2_ (HZO) /La_0.67_Sr_0.33_MnO_3_/SrTiO_3_ ferroelectric memristor. The device offers high stability, such as multi‐stage stable storage states (16‐state retention time can exceed 10^4^ s), high endurance performance (10^8^ cycles), and stable pulse modulation. At the same time, the device has an ultra‐low energy consumption of 121 fJ. In addition, the HZO memristor is capable of a wide range of synaptic behaviors and logic operations. Importantly, this work is the first to apply a reservoir computing network based on HZO memristors to the field of biological genetics. The network successfully achieves a biological codon recognition accuracy of over 97% via the dual‐feature strategy. This work provides concrete system and design ideas for achieving low‐cost and high‐accuracy codon recognition in the biological field.

## Graphical abstract

This work reports on epitaxially grown Hf_0.52_Zr_0.48_O_2_ ferroelectric memristors with high stability (high durability exceeding 10^8^ cycles and a retention time exceeding 10^4^ s for 16 states) and ultra‐low operating energy consumption (∼121 fJ). A memristor array‐based reservoir computing system is constructed and applied for the first time in the field of biological genetics, achieving a codon recognition accuracy rate exceeding 97%. This work reveals the immense potential of high‐performance hafnium‐based devices for applications in the field of biological genetics.

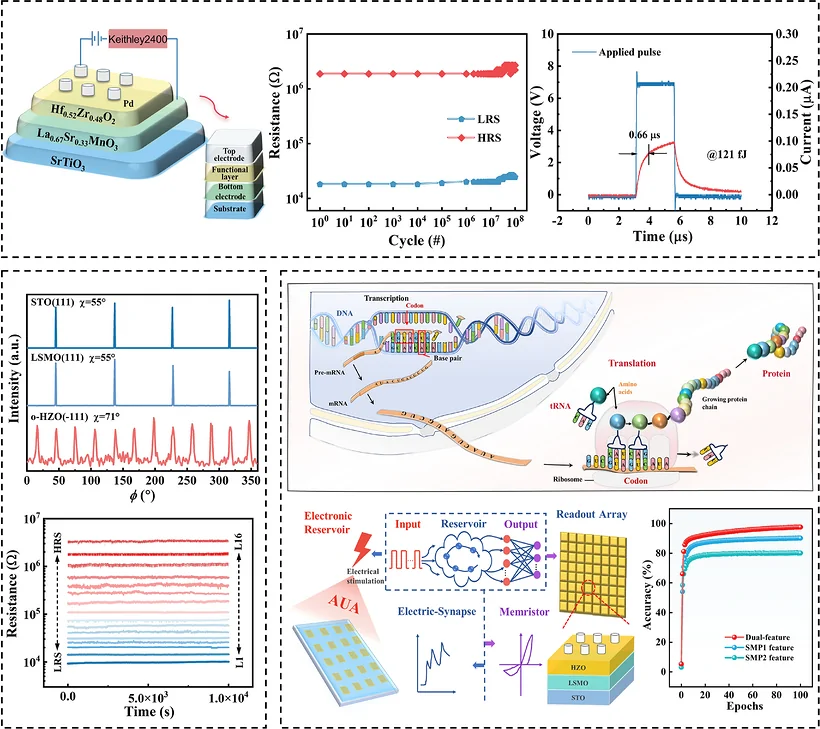

## 1 Introduction

With the rapid development of the era, the traditional von Neumann architecture of the computing system in the processing of massive information, showing certain technical limitations, its storage speed is far lower than the speed of computing, and gradually unable to meet the growing demand for data obtaining and storage [1, 2, 3, 4, 5, 6]. The neural system of the human brain is able to achieve memory storage of data from multiple sources through the computing in memory mechanism, which demonstrates higher efficiency and information integration ability [7]. Inspired by the human brain, neuromorphic computing is one of the most competitive candidates for traditional computer systems because of its potential to overcome the bottlenecks of the von Neumann architecture [8, 9, 10]. Neuromorphic computing architectures are implemented by simulating the function of neurons and synapses at the device or circuit level, but circuit‐level implementations typically require complex circuits to support information transmission, which can lead to additional energy consumption and latency [11]. Consequently, the development of artificial synaptic devices that can mimic the structure and working mechanism of biological synapses is pivotal [12].

In recent years, memristors have become one of the most promising artificial synapse candidate devices for constructing next‐generation computer architectures and realizing neuromorphic computing [13, 14, 15, 16, 17, 18]. With the gradual development of memristors, a variety of material systems (including transition metal oxides [19, 20, 21], sulfur compounds [22, 23], etc.) have been widely studied to achieve the resistive effect. However, ideal materials for memristors need to simultaneously meet many requirements such as high performance (including fast response speed, low operating energy consumption, multi‐level storage, long durability, terrific retention characteristics) and high reliability. Traditional memristors often face challenges: they rely on ion migration to form conductive filaments. This mechanism is susceptible to inherent defects, leading to randomness or instability in filament formation, which in turn causes fluctuations in switching characteristics (increased operating energy consumption) and reliability issues [19, 22]. These issues severely limit their application in the construction of high‐performance, high‐reliability neuromorphic systems. In contrast, the HfO_2_‐based ferroelectric memristor has attracted many researchers due to its excellent polarization controllability, high response speed, ultra‐low energy consumption, and multi‐value storage [16, 24, 25, 26, 27, 28]. More importantly, their resistance state switching depends on ferroelectric polarization reversal rather than ion migration, so no electroforming process is required, fundamentally ensuring the stability of the resistance state. These excellent properties allow for a wide range of applications in a variety of fields [1, 16, 29]. But a key challenge is that in most reported hafnium‐based films, the material is polycrystalline and often contains a second non‐ferroelectric phase, the monoclinic phase, m phase [30, 31]. Research indicates that the presence of non‐ferroelectric phases (such as the m phase) significantly degrades the overall performance of ferroelectric memristors, including their durability [16]. To address this, this study will attempt to prepare high‐quality hafnium‐based ferroelectric thin films using epitaxial growth technology. By controlling the crystal orientation of the film, it is expected to effectively suppress the formation of non‐ferroelectric phases (especially the m phase), reduce structural defects such as grain boundaries, and significantly enhance the durability of the resulting ferroelectric memristor devices.

One of the important tasks of neuromorphic computing is to understand and simulate the efficient information processing behavior of biological nervous systems [32]. Reservoir Computing (RC) network based on memristors have attracted widespread attention in this field due to their unique advantages. First, this method can directly process detected neural signals, thereby providing a better understanding of the nervous system. Second, compared to traditional neural networks, RC only requires training the readout layer, which significantly reduces the time cost of training [33]. Furthermore, the performance of RC network highly depends on the richness and stability of the storage pool state, which in turn directly depends on whether the memristors, as the physical foundation, can achieve stable and controllable state regulation [11, 34, 35]. Additionally, the low‐energy consumption characteristics of memristors make RC network based on memristors promising for achieving higher overall energy efficiency. Hafnium‐based ferroelectric memristors, with their multi‐level storage states, ultra‐low energy consumption, and excellent stability, perfectly align with the key requirements of RC network for devices. However, despite the significant potential of hafnium‐based memristors, their application research in RC network remains relatively limited [36]. On the other hand, although RC network have been proven to have good application prospects in field such as facial recognition, fingerprint recognition, and gesture recognition, there is still insufficient exploration of their application in areas requiring efficient biological gene recognition [11, 37, 38]. Here, it is hoped that an attempt will be made to achieve energy‐efficient bio‐codon recognition with high recognition rates using Zr‐doped HfO_2_ memristor with high stability, ultra‐low operating energy consumption, and polymorphism.

In this work, the device was epitaxially grown with highly oriented Zr‐doped HfO_2_ (HZO) ferroelectric films through single‐crystal substrate SrTiO_3_ (STO) and La_0.67_Sr_0.33_MnO_3_ (LSMO) buffer layers, with a ferroelectric domain flip angle of 180°. In addition, the HZO memristor devices have better multi‐level state (16‐state or 4 bit) retention performance (can exceed 10^4^ s), higher endurance (10^8^ cycles) and lower energy consumption (∼121 fJ). HZO‐based memristors can implement various synaptic behaviors, including paired‐pulse facilitation (PPF), post‐tetanic potentiation (PTP), and synaptic time‐dependent plasticity (STDP). The device can also perform logic operations for additive and multiplicative exchange laws by modulating the synaptic weights. In particular, a dual‐feature strategy is adopted to learn English alphabets for primary cognitive tasks by constructing an HZO ferroelectric memristors RC network. Based on the situation, the first application in the field of biological gene expression has been carried out. And we have successfully achieved more than 97% accuracy of biological codon recognition using HZO ferroelectric memristors RC network, which has the advantage of low cost and high efficiency. This work demonstrates the potential of hafnium‐based ferroelectric memristors for a wide range of applications in the field of achieving low‐cost and highly accurate codon recognition biology.

## 2 Results and Discussion

**FIGURE 1:**
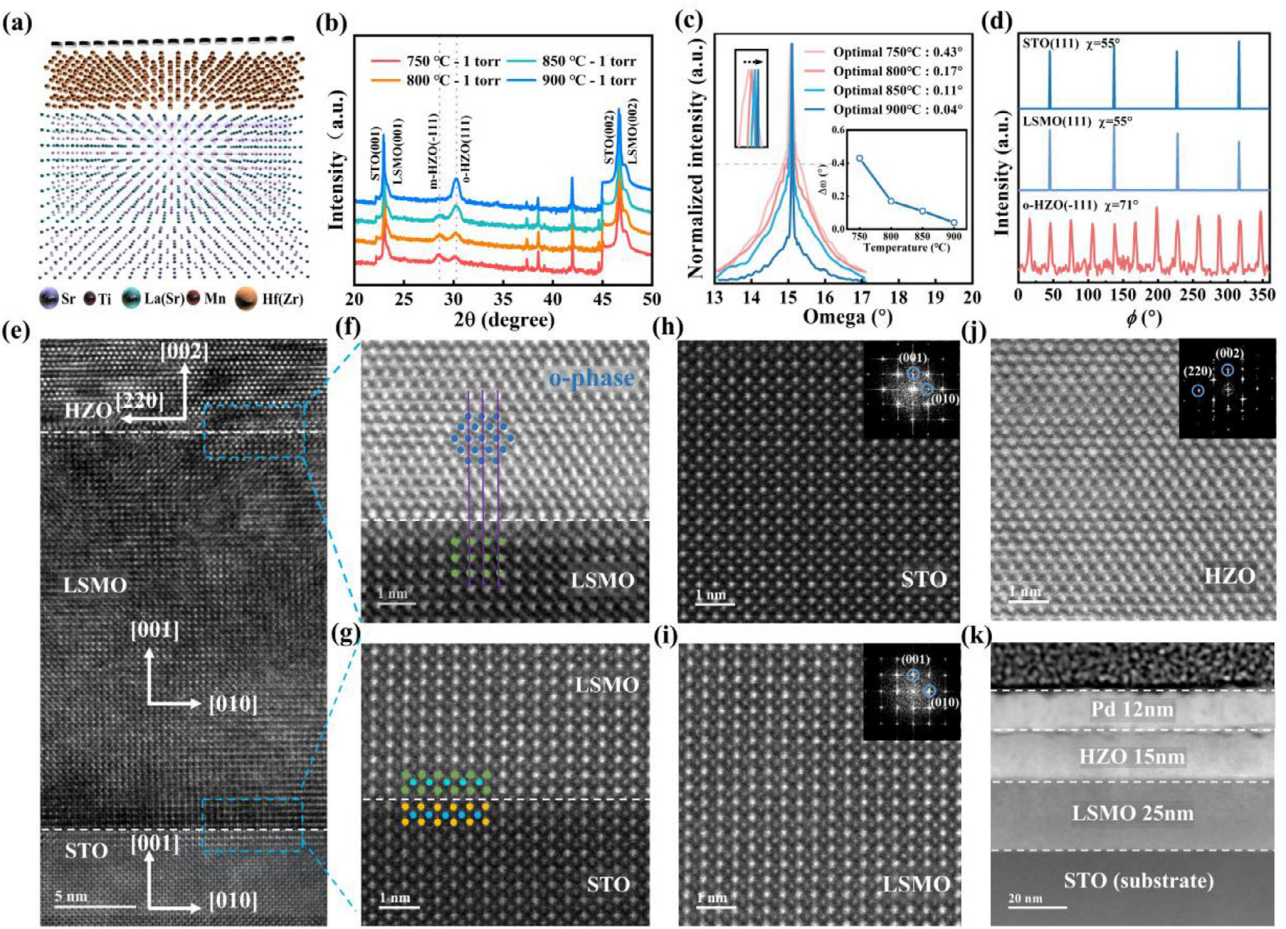
(a) Schematic diagram of the Pd/HZO/LSMO/STO device structure. (b) XRD at different temperatures under 1 torr annealing condition. (c) Rocking curves of HZO (111) peaks prepared at different temperatures under 1 torr annealing condition. (d) φ scan of STO, LSMO, and HZO (tested for best‐conditioned device). (e) High magnification image of HZO/LSMO/STO epitaxial structure device. (f) shows a high‐resolution image of the HZO layer with the LSMO bottom electrode layer. (g) shows a high‐resolution image of the interface between the LSMO bottom electrode layer and the substrate STO. (h) shows a high‐resolution image of the substrate STO along with the FFT of the region. (i) shows a high‐resolution image of the LSMO layer along with the FFT of the region. (j) shows a high‐resolution image of the HZO ferroelectric film along with the FFT of the region. (k) Low magnification image of the overall structure of the device.

Figure 1a illustrates the schematic diagram of the extrinsic structure of the HZO ferroelectric memristor. In this work, LSMO bottom electrode, HZO functional layer, and Pd top electrodes were grown on STO substrate. First, we fixed the annealing environment at 1 torr and the growth oxygen pressure at 97.6 mtorr, then investigated the effects of different growth temperatures (750°C, 800°C, 850°C, and 900°C) on HZO ferroelectric functional layer films. The x‐ray diffraction (XRD) results of HZO thin films are shown in Figure 1b. The different diffraction peaks observed at 27° to 32° correspond to the monoclinic crystal phase (m‐phase) (‐111) and the ferroelectric phase (o‐phase) (111) of the HZO ferroelectric films. The results show that the diffraction peak of the ferroelectric phase O‐phase (111) phase of the HZO film at around 30° gradually becomes larger as the HZO film growth temperature increases, while the m‐phase (‐111) gradually decreases at around 28°. It indicates that the crystalline quality of the film becomes better as the temperature increases, which is in line with the results of the previous studies that the crystallinity of the film grown at higher temperatures is better [16]. Figure 1c compares the rocking curves of the HZO films (111) crystalline phase grown at four temperatures, and the left inset shows that the rocking curves of the crystalline phase of (111) appear to be shifted to the right as the temperature increases, which is consistent with the phenomenon in XRD. The right inset shows the full width at half maximum dot‐line plots of the HZO films (111) peaks prepared at four temperatures, and it can be found that the half‐peak width of the HZO film grown at 900°C is the smallest (∼0.04°), this result indicates that the epitaxial HZO film grown at the growth temperature of 900°C exhibits a larger transversal coherence and smaller out‐of‐plane crystalline tilt distributions, which is even more validates the above conclusion [39]. In summary, HZO devices exhibit optimal crystalline quality under growth conditions of 900°C. In the following work, all tests on HZO ferroelectric devices were performed on device prepared at optimal conditions. In order to study the epitaxial growth relationship of the HZO in this work, a φ scan of the device was performed, as shown in Figure 1d. The results indicate that HZO exhibits twelve diffraction peaks at χ = 71°, while LSMO and STO each display four diffraction peaks at χ = 45°. For HZO with an orthorhombic structure, its (111) reflection should exhibit triple symmetry. Combining experimental results, it can be inferred that LSMO films are epitaxially grown on STO substrates in a cube‐on‐cube configuration. The (111)‐oriented o‐HZO lattice matches the LSMO lattice through four sets of rotations (0°, 30°, 60°, and 90°), thereby generating a 12‐fold symmetry peak [40, 41].

Subsequently, we performed microstructural characterization of the device. to analyze its physical structure. Figure 1e illustrates a high‐resolution scanning transmission electron microscopy (STEM) image of the cross‐section of the HZO/LSMO/STO structure, which provides a clearer view of the high‐quality epitaxial structure. This work employed (001)‐oriented STO single‐crystal substrates, upon which LSMO was epitaxially grown as the bottom electrode. The LSMO film grew with a plane oriented along the [001] direction, and HZO film was epitaxially grown along the [002] orientation, perpendicular to the plane. High‐resolution images of the HZO/LSMO interface and the LSMO/STO interface were obtained by zooming in on the interfaces at the two boxes in Figure 1e as shown in Figure 1f, g. Figure 1f illustrates a high‐resolution image of the HZO functional layer with the LSMO bottom electrode, where the HZO ferroelectric phase O‐phase has a well‐defined atomic arrangement and the highly oriented directional relationship is confirmed. The HZO layer exhibits an epitaxial relationship with the LSMO bottom electrode. A high‐resolution image of the interface between the LSMO layer and the STO layer is illustrated in Figure 1g, where a clear arrangement of atoms can be seen at the interface due to the same growth orientation of STO and LSMO, which proves the epitaxial growth relationship between the two layers. Figure 1h, i illustrate the Fast Fourier Transform (FFT) results of STO and LSMO, the crystal orientation of the two layers is consistent. The LSMO layer is exhibited co‐planar epitaxy, satisfying the out‐of‐plane [001] LSMO // [001] STO relationship, whilst in‐plane [010] LSMO // [010] STO, which further proves the good epitaxial relationship between the two layers. Figure 1j shows the FFT results of the HZO ferroelectric thin film. Through literature review and simulations using the unit cell for FFT and TEM‐Selected Area Electron Diffraction (SAED) analysis, confirm that HZO exhibits an out‐of‐plane orientation of [002] HZO // [001] LSMO along the growth direction, while in‐plane orientation is [1, 2, 3, 4, 5, 6, 7, 8, 9, 10] HZO // [100] LSMO [42, 43, 44]. Furthermore, to further demonstrate that this work pertains to the o‐phase rather than the easily confused T‐phase, a detailed comparison of the two lattice models was conducted, as shown in Figure S1. Analysis and comparison confirm that the HZO for this work is the O phase. These results confirm the high‐quality growth of the HZO/LSMO/STO films, thus, explaining well the above device performance. Figure 1k shows a low‐resolution image of the cross‐section of the overall structure of the Pd/HZO/LSMO/STO device, where the thickness of the Pd top electrode/HZO ferroelectric functional layer/LSMO bottom electrode layer is 12 nm/15 nm/25 nm, respectively, and the STO serves as the substrate of the device. The results of the energy spectrum analysis (EDS) of the device structure are given in Figure S2, where all the chemical elements of the HZO ferroelectric memristor were detected and analyzed, with the elements uniformly distributed in the structure. The results of the above work show that the HZO‐based ferroelectric memristors have a better epitaxial growth structure and the HZO films are also shown to have better crystallinity. These excellent results allow the HZO films of this work to be used as stable and durable storage media.

**FIGURE 2:**
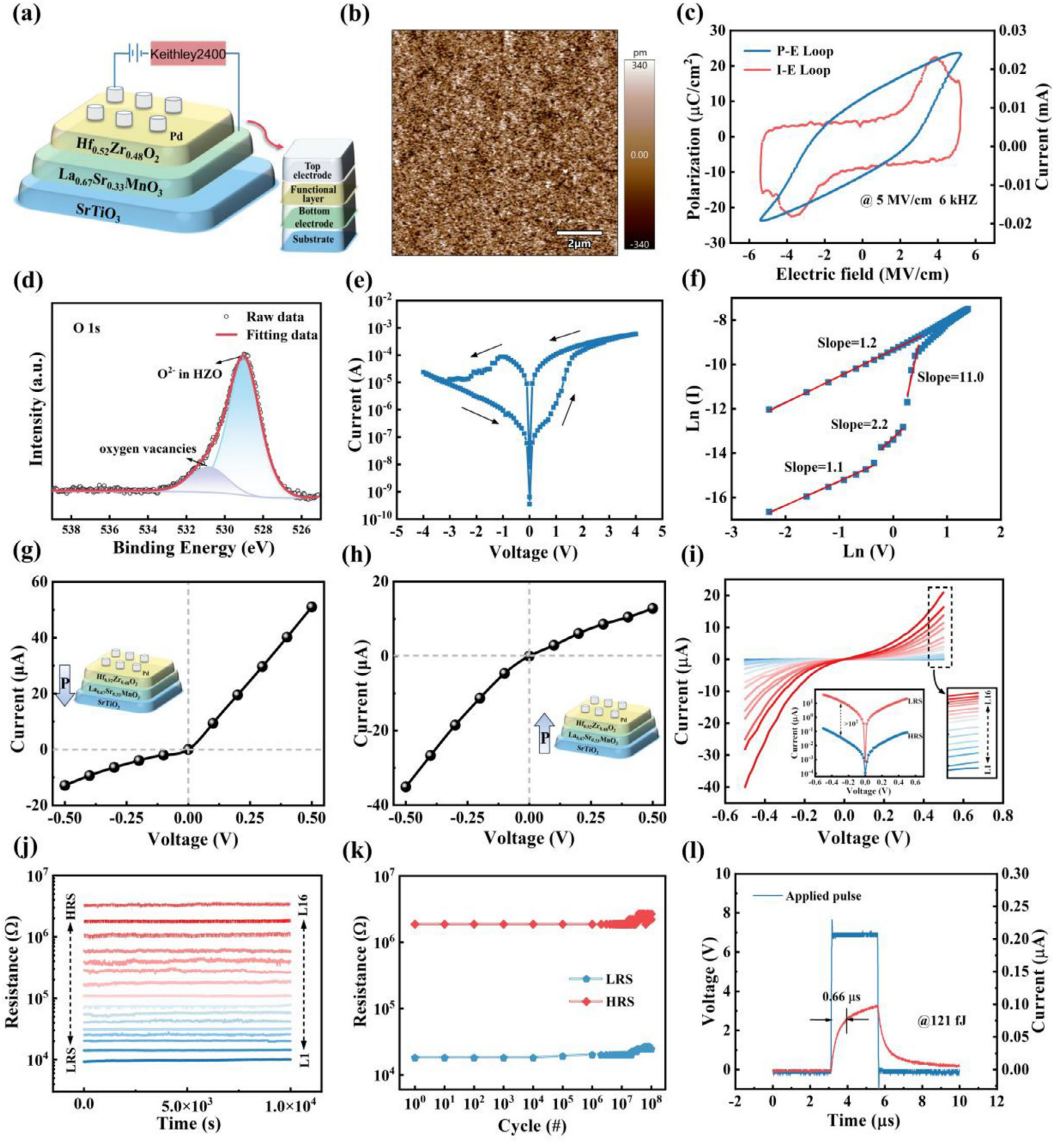
Characterization of the memristor with a Pd/HZO/LSMO/STO structure. (a) Schematic of the structure of the Pd/HZO/LSMO/STO memristor device. (b) AFM characterization image of the HZO‐based memristor at 10 µm × 10 µm region. (c) Typical ferroelectric hysteresis loop of HZO film. (d) XPS test of O1s energy level of HZO films. (e) I‐V curve obtained by applying 4 V scanning voltage under DC conditions. (f) Mechanism‐based fitting of I‐V curves for HZO device. (g,h) Represent the device's resistive state during read operations that control the ferroelectric polarization direction. (i) Testing of 16 resistance states of HZO ferroelectric memristors. (Varying V^−^ from −2.4 to −8 V, decreasing in 0.4 V steps, while maintaining a pulse duration of 50 µs and V^+^ at +6 V). (j) Retention characteristics of the 16 states of the HZO‐based memristor. (k) Endurance performance of the device up to 10^8^ cycles. (l) Response speed and energy consumption testing.

Following the investigation of optimal conditions, we conducted further testing on HZO devices fabricated under conditions of 900°C, 97.6 mtorr, and 1 torr. Figure 2a illustrates the electrical test setup based on HZO device and schematic diagram of the extrinsic structure of the HZO ferroelectric memristor. First, in order to check the growth quality of the film, the morphology was scanned using an atomic force microscope (AFM) over an area of 10 µm × 10 µm, and the result is shown in Figure 2b. The root‐mean‐square (RMS) roughness of the film reached a small value of 125.54 pm, and the scanning results of the devices prepared under other conditions are shown in Figure S3. In order to further characterize the ferroelectric properties of the device, using piezoelectric microscopy (PFM) tested the 10 µm × 10 µm region. Figure S4 illustrates the PFM images at different temperatures, where the ferroelectric domain flip angle increases with increasing temperature and the color contrast is more pronounced for the same bias applied. At 900°C, the HZO film exhibits a flip angle approaching 180°. The ferroelectric performance of the HZO ferroelectric device with optimum conditions was tested. Figure 2c illustrates a typical ferroelectric hysteresis loop curve (PE‐Loop) and the corresponding I‐E curve with 2Pr values up to 21.92µC/cm^2^ and 2Ps values up to 47.12µC/cm^2^. The PE‐Loops tested at different amplitudes as well as at different frequencies are given by Figure S5. In addition, the states of the chemical elements of the HZO film were analyzed using x‐ray photoelectron spectroscopy (XPS). The binding energies of the other elements are initially calibrated using the C 1s reference peaks. The XPS model of O 1s is given in Figure 2d, which clearly shows two prominent peaks, one corresponding to the lattice oxygen in HZO, and the other peak is the oxygen vacancy peak due to the presence of a large number of defects at the interface caused by lattice mismatch, located at 529.03 and 530.88 eV, respectively. The results of the XPS full spectrum analysis of the HZO film, the binding energy spectra of the Hf 4f and the Zr 3d peaks are illustrated in Figure S6. In summary, it can be concluded that the O 1s peaks of the HZO ferroelectric films prepared in this work are dominated by lattice oxygen, indicating that the HZO films have high crystallinity and chemical stability [45, 46, 47, 48].

Next, the electrical performance of the HZO device was characterized in order to investigate the resistive switching characteristics of the HZO ferroelectric device. The 4 V direct current (dc) voltage was applied to the top electrode of the device, and the bottom electrode is grounded. As shown in Figure 2e, the device exhibits bi‐directional modulation behavior. The obtained I‐V curves were analyzed via fitting, as shown in Figure 2f. The results indicate that the fitting of the high‐resistance state (HRS) region is consistent with the Space Charge‐limited Current (SCLC) mechanism, while the fitting of the low‐resistance state (LRS) region is consistent with the Ohmic mechanism [49, 50, 51, 52]. Subsequently, we modulated the ferroelectric orientation using pulses: first, a +8 V pulse was applied to shift the polarization downward, followed by a ±0.5 V voltage pulse to read out the low‐resistance state. Next, a reverse voltage pulse was applied to shift the polarization upward, and the state at this point was read out. As shown in Figure 2g, h, this demonstrates that the ferroelectric material can modulate the diode behavior [53]. Collectively, these results verify that the resistive switching of our device is dominated by reversible ferroelectric polarization modulation, with trace interfacial oxygen vacancies serving as an auxiliary factor that mediates the observed SCLC and Ohmic transport behaviors. The multilevel storage states of the HZO‐based device were then tested, as shown in Figure 2i, which demonstrates 16 storage states. The figure below shows the switching ratio of the device. Switching ratios can exceed two orders of magnitude. Then, the 16 obtained states were tested for retention performance, as shown in Figure 2j, and each resistor state showed a weak fluctuation within 10^4^ s, showing a better stability. Furthermore, the device was subjected to an endurance test as shown in Figure 2k. In order to ensure the accuracy and reliability of the data, a total of 107 data points were collected over 10^8^ cycles, and the test results show that the HZO device exhibits excellent stability performance. Additionally, the response speed and energy consumption of the device were tested (Figure 2l), and the testing process is illustrated in Figure S7. This device can achieve a low operating energy consumption of 121fJ, laying a certain device foundation for realizing low‐energy brain chips. Supplementary Information Tables S1 and S2 provide a performance comparison of individual devices. In this study, HZO ferroelectric memristors grown by epitaxial growth showed greater advantages in energy consumption, multi‐level storage, retention, and durability compared to other hafnium based and non‐hafnium‐based ferroelectric devices. These stable resistive switching behaviors of HZO‐based ferroelectric based on epitaxial growth enable them to exhibit higher fitness for storage applications requiring high stability and multiple states.

**FIGURE 3:**
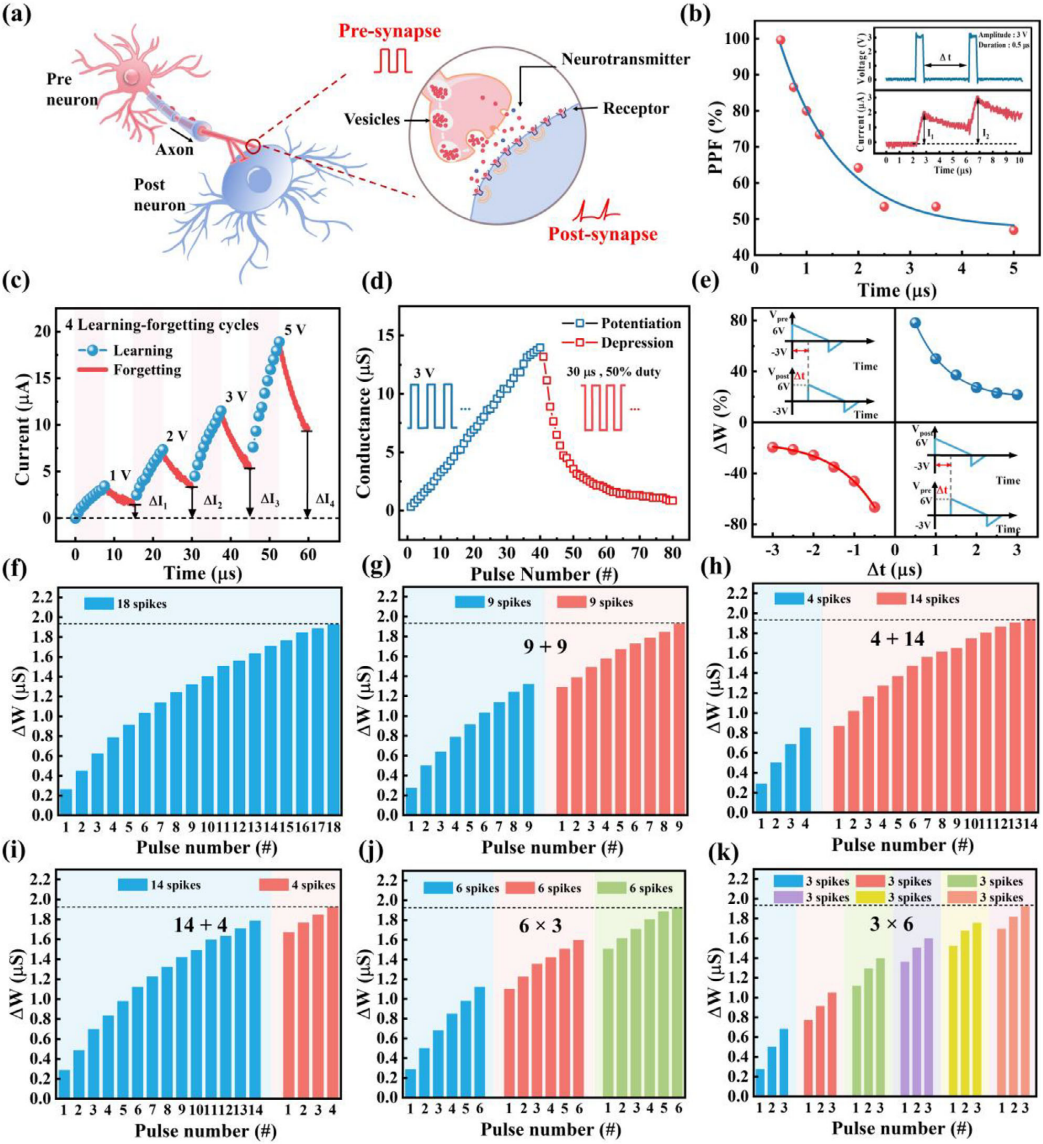
Characterization of artificial synaptic properties of HZO‐based memristors. (a) Schematic representation of neuronal and synaptic structures in the brain. (b) PPF index: two pulses with an amplitude of 3 V and a duration of 0.5 µs were applied, with the interval increasing from 0.5 µs to 5 µs. (c) Simulation of learning and forgetting four times in a row (“learning” pulse parameter: 0.75 µs, 75% duty, 1∼5 V). (d) Shows the conductance value change curve of HZO ferroelectric memristor based on 40 successive same amplitude pulse stimuli. (40 pulses each for positive and negative directions, total time of 30 µs, 50% pulse duty). (e) STDP characterization. (f) The relationship between absolute weight and the number of stimulus pulses. (Pulse amplitude: 4 V, pulse duration:100 ns). (g) Verification of linear relationship. (h,i) Addition operation and addition exchange law. (j,k) Multiplication operation and multiplication exchange law.

In the human brain system, the transmission of information is carried out by synapses, which are responsible for transmitting stimuli from one neuron to another, as shown in Figure 3a [54]. Pre‐synaptic neurons respond to stimuli and release neurotransmitters for transmission to post‐synaptic neurons. Synaptic weights represent the strength of signals transmitted by synaptic connections between neurons and are a key factor in synaptic plasticity. Adjusting synaptic weights can play an optimizing role for neural networks because it improves the accuracy and efficiency of neural network operations, making them better suited to various artificial intelligence tasks. In order to investigate the artificial synaptic properties of the HZO memristors, a series of pulse modulation tests were performed. Pulses of different amplitudes, intervals, and durations were first applied to the device, as shown in Figure S8. And it can be observed that the device conductance values increased with higher pulse amplitudes, longer pulse durations, and shorter pulse intervals. Figure S9 simulates the memory recognition process of the uppercase English letter “Y”, which becomes darker and darker as the number of applied pulses increases, thus revealing the phenomenon that the conductance value of the HZO memristor device increases with the number of pulses. After exploring the series of laws, the conductance changes of the HZO devices were further analyzed, and it was found that HZO‐based memristor devices can mimic several important functions of synapses, similar to some functions of the human brain system. As shown in Figure 3b, the top right inset shows a schematic diagram of paired pulses with an amplitude of 3 V and a duration of 0.5 µs applied to the HZO device to simulate PPF by varying the interval between the two stimulus pulses. PPF index means that when two consecutive pulses are applied, the magnitude of the response of the second pulse will outweigh that of the first, and depends on the time interval between the two pulses. The simulation results can be expressed by the following equation:PPF  =  (I_2_ − I_1_)/I_1_ × 100%, where I_1_ and I_2_ denote the maximum postsynaptic current values after the first and second stimuli, respectively. The fit results are in better agreement with the measured values. In addition, the PPF index of pulses with different amplitudes were also explored, and as shown in Figure S10a,b, it is clear that increasing the stimulus pulse amplitude progressively increases the synaptic weights. Figure S10c,d give the post‐tonic potentiation (PTP) index versus interval. Similarly, applying two sets of differently spaced pulses to the device achieved a change from PPF to double‐pulse suppression (PPD) index, as shown in Figure S11. Additionally, the device was simulated to undergo four cycles of learning and forgetting (as shown in Figure 3c), achieved by applying voltage pulses of 1, 2, 3, and 5 V in succession. When subjected to four successive voltage pulses of varying amplitudes, the device exhibited progressively larger response currents during identical forgetting periods(ΔI_4_ > ΔI_3_ > ΔI_2_ > ΔI_1_), indicating a gradual increase in memory strength. This process mirrors the human brain's cycle of learning, forgetting, and relearning. The effect of slowing down the memory forgetting was achieved by increasing the relaxation time via changing the pulse parameters, which also reflected the memory retention enhancement of the HZO memristor device, as shown in Figure S12. Based on the memory properties of the HZO memristor device, we successfully simulated the Pavlovian conditioned reflex experiment as shown in Figure S13. There are other forms of synaptic plasticity, and Figure 3d illustrates LTP and LTD properties obtained by applying a pulse sequence of the same amplitude. It also proved that the device had excellent bi‐directional regulation characteristics. Figure 3e illustrates the simulation of STDP, which refers to the fact that the synaptic weights change with different intervals between the pre‐synaptic and post‐synaptic membranes receiving stimulation. When the presynaptic membrane receives stimulation before the postsynaptic membrane, the prominence weight increases slowly as the two intervals become larger; when the postsynaptic membrane receives stimulation before the presynaptic membrane, the prominence weight decreases slowly as the two intervals become larger. Besides, three other typical learning rules including pulse rate dependent plasticity (SRDP), pulse width dependent plasticity (SDDP) and pulse amplitude dependent plasticity (SADP) were also simulated, as shown in Figure S14.

The decimal arithmetic function was developed from counting, and it has been demonstrated that memristor devices can emulate this logic calculation [1]. It has found that conductance modulation driven by positive / negative pulses exhibits high linearity and symmetry, making it highly suitable for decimal operations. In this work, the synaptic weights of HZO memristors can be periodically adjusted by applying positive pulses, enabling computational behavior based on synaptic weight changes. ΔW denotes the absolute weight, expressed as ΔW = W_n_‐W_0_, where W_n_ represents the synaptic weight after applying n pulses. As shown in Figure S15, when 18 consecutive positive pulses (+4 V, 100 ns) were applied, the device exhibited a linear change in conductance (Figure 3f). And Figure 3f achieves ΔW of ∼ 1.92 µS. Subsequently, for calibration purposes, two sets of pulses were continuously applied to verify the performance of the device. As shown in Figure 3g, the final ΔW of the device gradually increased to approximately 1.92 µS. In the subsequent calculation, when the absolute weight increased to 1.92, it was indexed as the decimal number 18. Figure 3h, i illustrates the commutative law of addition. By applying two consecutive pulse sequences separately, the weight value of ΔW_18_ approximates to 1.92, which proves the addition of “14 + 4 = 18” and “4 + 14 = 18”, as well as the addition commutative law of “14 + 4 = 4 + 14”. Based on the logic for implementing addition, the calculation logic for multiplication is realized, as shown in Figure 3j. When a continuous sequence of three groups of pulse stimuli (each group consisting of 6) was applied to the device, the final ΔW_18_ value showed a weight of approximately 1.92, indicating that the addition logic calculation of “6 + 6 + 6 = 18” had been completed. And this precisely completes the multiplication logic of “6 × 3 = 18”. Similarly, when applying six consecutive pulse stimuli, the multiplication calculation of “3 × 6 = 18” was achieved. Therefore, the multiplication commutative law “6 × 3 = 3 × 6” has been achieved.

**FIGURE 4:**
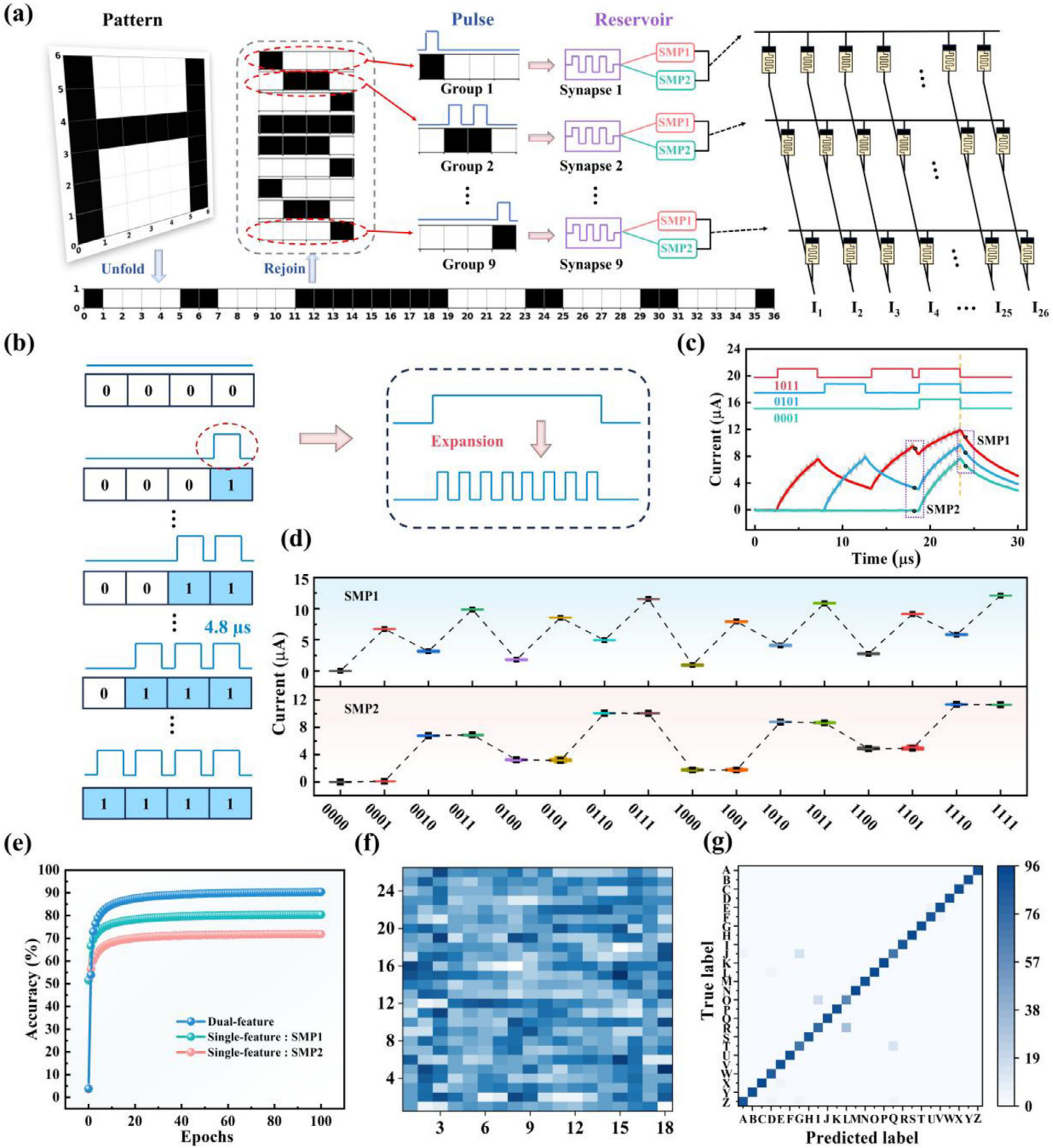
Alphabet recognition based on HZO ferroelectric memristor RC network. (a) Schematic of the working principle of the HZO ferroelectric memristor‐based RC network used to recognize the 26 letters of the alphabet. (b) Schematic diagram of four‐digit electrical pulse input from “0000” to “1111” based on binary numbers, with the illustration on the right indicating that each large pulse consists of eight small pulses. (c) Input waveforms of three types—“0001”, “0101” and “1011”—were plotted, along with the response curves from actual tests showing current variation over time. (d) Statistical results of 20 repetitive actual tests of two feature outputs on 16 types of inputs. (e) Evolution of single‐feature and dual‐feature based accuracy during readout network training. (f) Readout layer weight map of the dual‐feature output. (g) Confusion matrix results obtained for the dual‐feature output.

HZO ferroelectric memristors offer new implementation solutions for RC network recognition applications thanks to their adjustable multiple states, ultra‐high stability, and ultra‐low energy consumption performance. In this work, an RC network for alphabet recognition was constructed based on the HZO device, as shown in Figure 4a, which consists of a synaptic storage layer of HZO memristor and a readout layer implemented by an array of devices. The 6 × 6 pixel image of the letter “H” is first flattened sequentially into a 36D feature vector, which is then converted into nine sets of temporal input signals via a four‐pulse coding scheme. All input electrical pulses adopt a fixed amplitude of 3 V and a uniform pulse width of 4.8 µs. These pulse signals are processed by the synaptic reservoir of the HZO memristor to generate corresponding single‐feature and dual‐feature outputs at two preset sampling moments (the first sampling point SMP1 and the second sampling point SMP2). Finally, the extracted feature signals are subjected to weighted training by a readout layer constructed via algorithmic simulation, where the multi‐conductance state characteristics of HZO memristors are utilized for model construction [11]. The significant advantage of this scheme is that only the readout layer needs to be trained, which significantly reduces the computational cost compared to full network training. The electrical pulse sequences are given by Figure 4b, showing five typical electrical pulse sequences of “0000”, “0001”, “0011” “0111”, and “1111”, and the duration of each large pulse is 4.8 µs. Since HZO devices tend to saturate under prolonged pulsed stimulation, we then used eight consecutive short‐term pulses to simulate a long‐term pulsed stimulus in this work, as shown in the vignette on the right. Figure 4c illustrates the current response of the HZO memristor device under stimulation with three different electrical pulse sequences. Despite the fact that the last pulse states are all high level “1”, the responses differ, confirming that the device has a pulse sequence‐dependent conductance memory effect. To ensure the distinguishability of the sampled data, the SMP1 shown in Figure 4c was chosen as the initial eigenvalue point in this study. Statistical results about SMP1 were obtained through 20 cycles of testing, as shown in the upper panel of Figure 4d. The tiny difference in the characteristic response of SMP1 for different electrical pulse inputs affects the differentiation of English letter recognition. Considering the above limitation of feature distinguishability, we adopted a dual‐feature sampling method, as shown in Figure 4c, where a SMP2 is selected in the third pulse region. The statistical results of the 20 repetitions of the test are shown in the lower panel of Figure 4d. All the current response plots of 20 repetitive tests obtained based on 16 electrical pulse sequences are shown in Figure S16, which further demonstrates the high stability of the HZO ferroelectric memristor device in this work. Furthermore, the scheme successfully achieves the efficient conversion of four‐bit pulse input to two‐bit feature output through the calculation of the memory storage layer, and maps it to the readout layer of the memory array for processing. In order to verify the superiority of the dual eigenvalue sampling method, we compared and evaluated the performance of English letter recognition under SMP1 single feature, SMP2 single feature, and dual feature extraction methods. As shown in Figure 4e, the recognition accuracies reach 80.4%, 71.8%, and 90.4%, respectively, and the dual eigenvalue approach has a faster convergence rate. (The loss functions of the three eigenvalue methods over 100 training cycles are shown in Figure S17) The final weights of the training used to recognize the 26 letters of the alphabet are shown in Figure 4f. The y‐axis represents the associated 26 letters of the alphabet, and the x‐axis represents the dimension of the reservoir state corresponding to the 18 data points (6 × 6/4 × 2) along the x‐axis. Figure 4g shows the confusion matrix for the 26 alphabets recognition test via the dual eigenvalue approach. These excellent test results prove that HZO ferroelectric memristors have great potential for applications in areas related to letter recognition.

**FIGURE 5:**
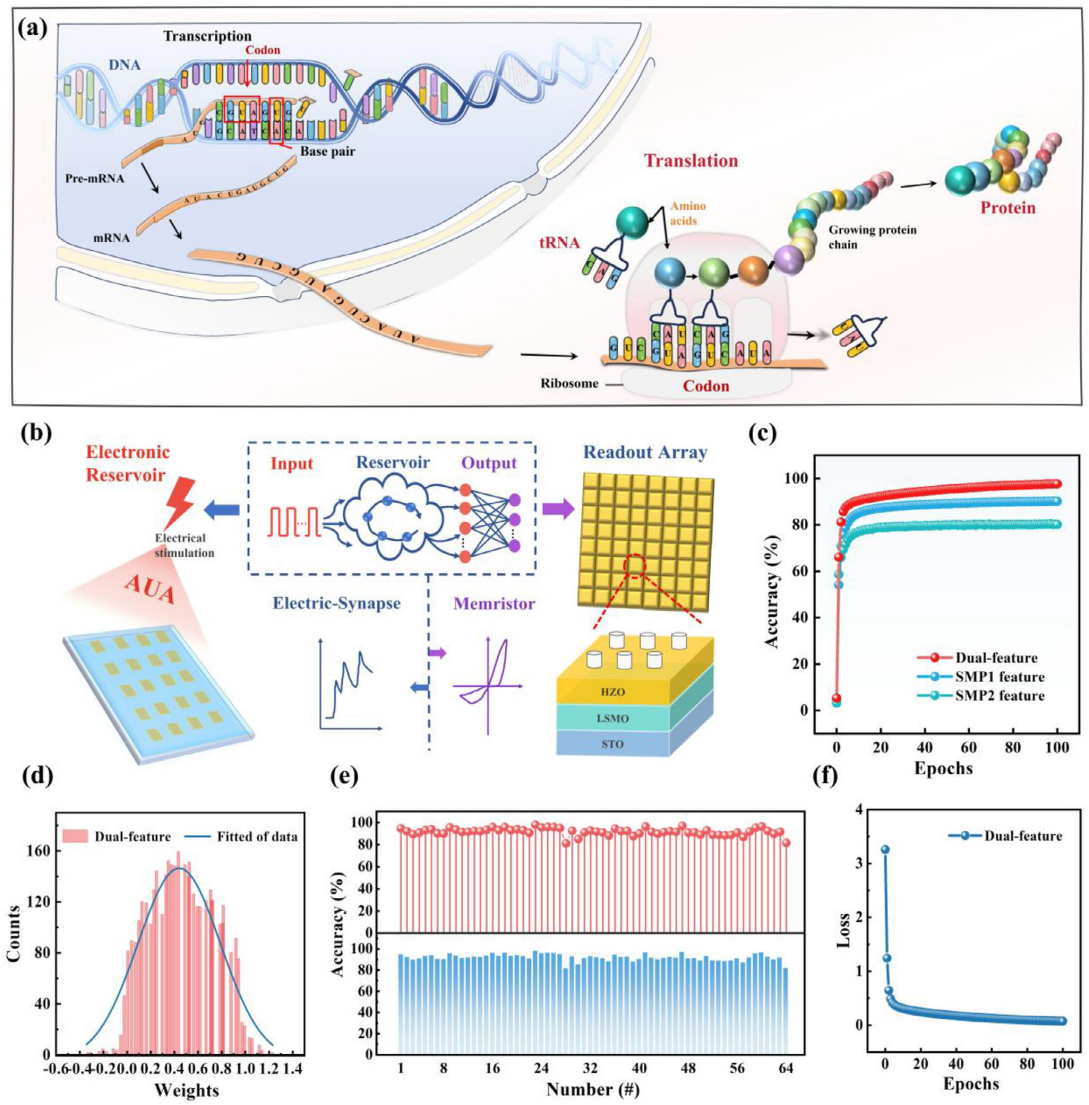
Codon recognition based on HZO ferroelectric memristor RC network. (a) Schematic diagram of the gene expression process in a biological system. Three neighboring bases determine a codon and each codon determines an amino acid. During translation, a protein with a specific amino acid sequence is synthesized by a tRNA carrying a codon that is recognized and paired with a codon on the mRNA. (b) Schematic of the HZO memristor RC neural network performing the codon identification task. The illustration in the dashed box shows the abstract RC neural network. The raw input pulse information is transferred into the reservoir, and the input is nonlinearly mapped to the feature output based on the EPSC response. Finally, the HZO memristor array receives the output of the reservoir and performs readout training. (c) Evolution curve of the recognition accuracy of the 64 codons obtained based on the three eigenvalue methods. (d) Statistical plot of simulated training weight values obtained using the dual‐feature approach. (e) Scatterplot as well as histogram distribution of recognition accuracy for each codon. (f) Loss evolution image.

Genes, the basic unit of inheritance of information about life, are mainly composed of DNA (deoxyribonucleic acid). It encodes and synthesizes proteins through specific sequences of DNA, which in turn play a key role in regulating the structure and function of organisms as well as their genetic characteristics. In 1953, scientists James Watson and Francis Crick proposed the double helix structure of DNA, successfully revealing the unique way in which DNA encodes information [55]. Gene expression is a complex process that begins with the transcription of DNA to form RNA (ribonucleic acid), which is then translated and synthesized into proteins, as shown in Figure 5a. It is worth noting that codon recognition plays an extremely important role in the translation process. Each codon consists of three adjacent nucleotides (A, U, C, G) that can encode specific amino acids or termination signals that direct protein synthesis. Of the 64 codons available (64 codons are shown in Figure S18), 61codons are used to encode 20 amino acids, and the remaining 3 codons are termination codons. Distinguishing all 64 distinct codons constitutes a high‐complexity multi‐classification task, far exceeding simple binary proof‐of‐concept tests, and this biological classification task is tightly coupled with the core physical characteristics of our HZO ferroelectric memristor: the volatile transient conductance dynamics of the device serve as the reservoir's feature extraction foundation, while its tunable non‐volatile multi‐level conductance states support weight mapping for readout classification. Codon recognition is central to the field of gene expression analysis, which not only helps researchers to gain a deeper understanding of gene function, but may also play an important role in predicting prokaryotic gene start sites [56] and assessing deleterious mutations in the human genome. Abnormal codon pairing and gene mutations will interrupt normal protein biosynthesis and induce hereditary diseases, metabolic disorders, and other pathological phenotypes, making high‐precision codon identification a prerequisite for auxiliary gene lesion diagnosis. However, current traditional codon recognition methods have limitations in accuracy and efficiency [57], making it difficult to meet the growing demand for genetic research. In contrast, this work innovatively uses HZO ferroelectric memristors to construct an RC network, which achieves high‐precision recognition of codons and provides a better solution for gene sequence interpretation and expression analysis. Compared with traditional methods, this RC network can handle complex genetic data more efficiently, demonstrating higher recognition accuracy and faster processing speed in the face of massive genetic information.

Inspired by the human brain system's capabilities in parallel processing, non‐linear mapping, and efficient feature extraction, a reservoir computing system based on HZO memristor was constructed and biological codons were learnt for cognitive tasks, as shown in Figure 5b. An electrical pulse stimulus representing the codon “AUA” encoding isoleucine is applied directly to the synaptic reservoir of the HZO memristor, and the signal output from the synaptic reservoir is weighted and trained by the memristor array. All electrical responses of the devices and reservoir‐state features SMP1 and SMP2 in this work are experimentally measured. Data preprocessing and the readout layer are simulated based on the measured device data, and a complete hardware array is not fabricated. Although the fully hardware‐based scheme delivers high integration and lower power consumption, it is constrained by device mismatch and complicated array control. Compared to other networks, the computational cost as well as the time consumption to achieve the recognition task can be significantly reduced. Figure 5c illustrates a dotted line plot of the evolution of accuracy with training rounds for codon recognition based on the three eigenvalue methods described above. The results of 64 codons recognized based on the dual eigenvalue method have faster convergence and higher recognition accuracy than the other two methods. Figure 5d illustrates a statistical plot of the simulated training weight values for the data obtained by the dual eigenvalue approach. Most of the weight values are concentrated in certain intervals (0.3–0.7), and the training is relatively stable, with no excessive dispersion of weight values or flooding of extreme values, which reflects the controllability of the training process to some extent. The specific recognition accuracies for each codon are illustrated by Figure 5e, with serial numbers substituted for the recognition tasks due to the fact that there are too many tasks to display clearly (as shown in Table S3). Sixty‐four codons were classified and identified, achieving an accuracy rate of 97.4%, showing a high level of accuracy. As shown in Figure 5f, the increase in recognition accuracy during training is also accompanied by a decrease in cross‐entropy loss.

By leveraging the unique synaptic resistive switching properties of epitaxial HZO ferroelectric memristors, this work bridges neuromorphic device physics and practical bioinformatics analysis, offering a low‐power hardware‐oriented prototype strategy for future on‐chip gene detection. In summary, HZO ferroelectric memristors have good potential for neural network computation and can be applied in the field of low‐cost and high‐precision codon recognition biology.

## 3 Conclusion

In this work, a ferroelectric thin film memristor with epitaxially grown structure of Pd/HZO/LSMO/STO was designed and studied in detail. HZO film‐based devices have high stability, including multiple levels of stable memory states (16 states, with each memory state holding time exceeding 10^4^ s), and high endurance (10^8^ cycles). In addition, these devices have lower energy consumption (121 fJ) and can simulate a variety of synaptic behaviors and logical operations by continuously modulating the conductance. This work develops the learning of cognitive tasks on the English alphabet as well as the codons that are important in the translation of biological genes by constructing a reservoir computing network based on HZO ferroelectric memristors. Using the dual‐feature approach, the system successfully achieves biological codons recognition accuracy of over 97%. These findings promote the further application of HZO ferroelectric memristors in the biological field and also highlight their great potential for application in neuromorphic computing.

## 4 Experimental Section

### 4.1 Fabrication of Pd/HZO/LSMO/STO Devices

In this work, HZO‐based ferroelectric memristor devices were prepared using pulsed laser deposition technique. First, 25 nm LSMO film was epitaxially grown on single crystal STO substrates at 750°C and 175.5 mtorr deposition oxygen pressure. Afterward, HZO films were grown on the LSMO layer using a Physik COMPex Pro 20S KrF excimer laser (λ = 248 nm) at 2 Hz at four different temperatures. And the cooling deposition process was carried out at a rate of 5°C/min. Finally, 50 µm of Pd top electrode was deposited using magnetron sputtering equipment at an argon flow rate of 25 Sccm.

### 4.2 Characterization and Measurement

X‐ray diffraction spectra were collected using a diffractometer in physical scanning mode. Thin film surface morphology and PFM tests were performed using a commercial AFM microscope (Cipher‐S, Asylum Research, GAI‐SA). XPS elemental analyses were tested using a Thermo Fisher ESCALAB Xi+. TEM and EDS tests were performed on the HZO devices using a Fei Tecnai G2 F20 ST FE‐TEM instrument. The electrical characteristics of the HZO memristor were thoroughly tested using a Keithley 2400 instrument, Keysight 33600A Arbitrary Function Generator and a RIGOL MSO5354 digital oscilloscope.

## Conflicts of Interest

The authors declare no conflicts of interest.

## Supporting information




**Supporting File**: advs76830‐sup‐0001‐SuppMat.docx.

## Data Availability

The data that support the findings of this study are available from the corresponding author upon reasonable request.
