# Supplementary material for: An Efficient Biological Codon Recognition Reservoir Computing System Based on Low‐Energy Epitaxial Hf0.52Zr0.48O2 Ferroelectric Memristors
Source: Adv Sci (Weinh). 2026 Sep 9:e00021. Online ahead of print. doi: 10.1002/advs.202600021 (PMC13555366; doi:10.1002/advs.202600021)
Supplement: Supplementary file 1 [file ADVS-9999-e00021-s001.docx]

**Supplementary Information**

**An efficient biological codon recognition reservoir computing system based on low-energy epitaxial Hf_0.52_Zr_0.48_O_2_ ferroelectric memristors**

Ying Liu^1,^*^#^*, Jikang Xu^1,^*^#^*, Yongqing Jia^1^, Wenxuan Wang^1^, Weifeng Zhang^1^, Biao Yang^1^, Xiaobing Yan^1,*^

*^1^ Key Laboratory of Brain-like Neuromorphic Devices and Systems of Hebei Province, College of Electronic and Information Engineering, Hebei University, Baoding 071002, P. R. China*

*^#^These authors contributed equally to the work*

*^*^Author to whom correspondence should be addressed: yanxiaobing@ime.ac.cn*

**Figure S1** (a)and (b) Atomic structures of the O phase and T phase HfO_2_. The yellow and red spheres represent the Hf and O atoms, respectively.

We compared the lattice models of the orthorhombic phase (O phase) with the Pca2_1_ space group and the tetragonal phase (T phase) with the P4_2_/nmc space group, as shown in **Figure S1**. These were rotated to align with the orientation corresponding to the HZO thin film imaged via TEM. By comparing the lattice arrangement schematic provided by TEM, the PFM polarization response, and ferroelectric property testing, we comprehensively determined that the sample indeed resides in the ferroelectric orthorhombic phase.

**Figure S2** Overall EDS analysis image of the HZO ferroelectric device.

**Figure S3** AFM characterization images of HZO thin films with different growth temperatures based on 97.6 mtorr deposition oxygen pressure and 1 torr annealing oxygen pressure condition. (a-c) Surface morphology of HZO thin film AFM prepared at 750 °C, 800 °C and 850 °C.

**Figure S4** Characterization of HZO thin films PFM with different growth temperatures based on 97.6 mtorr deposition oxygen pressure and 1 torr annealing oxygen pressure condition. (a-d) PFM flip angles of HZO thin films prepared at 750 °C, 800 °C, 850 °C and 900 °C.

**Figure S5** Ferroelectric hysteresis loop test. (a, b) P-E loop and corresponding IV plots for different voltage amplitude tests at 10 kHz frequency. (c, d) P-E loop and corresponding I-V plots for different frequency tests.

**Figure S6** (a) XPS full spectrum analysis results of HZO thin films. (b) XPS binding energy spectrum of Hf 4f. (c) XPS binding energy spectrum of Zr 3d.

**Figure S7** Schematic diagram of the response speed test circuit.

The device underwent response speed and energy consumption testing. First, single-pulse stimulation tests were performed on the device using a pulse signal generator, as shown in **Figure S7**. The total pulse from the pulse generator is acquired via CH1 channel, while the response voltage across Rs is acquired via CH2 channel. Thus, the device's voltage drop V_m_ = V_CH1_ - V_CH2_, and current I = V_CH2_ / R_s_, where R_s_ = 60 MΩ. Specifically, a 7 V square wave voltage with a duration of 2.5 μs is applied to the device. This single-pulse stimulus activates the HZO device. The voltage value across Rs is recorded. Subsequently, the time interval between pulse application and the HZO device reaching its fully activated state is recorded; this interval is employed for integration calculations. Energy consumption may be obtained by integrating instantaneous power over time, expressed as: W = $\int_{t1}^{t2} P\left( t \right)dt=\int_{t1}^{t2} U\left( t \right)*I\left( t \right)dt$. To calculate energy consumption, multiply the device's voltage V_m_ by its current I. The product is plotted as a two-dimensional function curve of instantaneous power versus time. Based on this function, perform numerical integration calculations on the identified time intervals. In summary, the energy consumption of this device is 121 fJ.^[1-2]^

**Figure S8** Effect of different electrical pulse parameters on device conductance values. (a,d) Effect of different amplitude (0.5,1,3,5 V) of electric pulse on the conductance value of the device, with the pulse duration and interval kept constant at 0.5 μs. (b,e) Effect of electrical pulses of different durations (0.25,0.5,0.75,1 μs) on the conductance value of the device with pulse amplitude of 3 V and interval of 0.5 μs kept constant. (c,f) Effect of electrical pulses with different intervals (0.25,0.5,0.75,1 μs) on the conductance value of the device with constant pulse amplitude 3 V and duration 0.5 μs.

**Figure S9** Recognition memory process for the letter “Y”. (a) The prepared HZO memristor device, and the inset shows the array area used for testing. (b) Schematic diagram of the testing of the letter “Y”. (c) Memory recognition process of the letter “Y” by applying 10, 15, and 20 cycles of duration 0.75 μs, interval 0.5 μs, and amplitude 3 V, respectively. The color of the letter “Y” is getting darker and darker.

**Figure S10** (a) Test results of the anterior and posterior two postsynaptic currents at different amplitudes. (Pulse duration: 0.5 μs; interval: 0.5 μs). (b) PPF index fitting results at different amplitudes. (c) Current responses at different intervals. (d) PTP index.

Post-tonic potentiation (PTP) index, a short-term form of enhancement of synaptic plasticity, refers to a temporary enhancement of synaptic transmission efficacy following high-frequency (tonic) stimulation of presynaptic neurons.^[3]^ It can be expressed by the following equation：$PTP=(I_{10}-I_{1})/I_{1}\times100\%$，where I_10_ and I_1_ each denote the postsynaptic current after the 10th and 1st stimulus pulses. A slow decreasing trend in the maximum postsynaptic current after the 10th stimulus as the pulse interval increases can be observed in **Figure S10c**. **Figure S10d** shows the PTP index versus time interval, and the synaptic weights can be increased by decreasing the pulse interval.

**Figure S11**. Test result from PPF to PPD by changing the pulse interval. (PPF: 30 pulses; 3 V; 0.5 μs duration; 0.5 μs interval; PPD: 10 pulses; 3 V; 0.5 μs duration; 2.5 μs interval.)

**Figure S12** Effect of varying pulse sequences on the EPSC of the HZO memristor. (a-c) Effect of different pulse amplitudes (0.5, 1, and 3 V) on EPSC, with the interval of 0.5 μs, duration of 0.5 μs, and number of pulses 40 kept constant. (d-f) Effect of different pulse durations (0.25, 0.5, 0.75 μs) on EPSC, with the interval of the pulses 0.5 μs, amplitude 3 V and number 40 kept constant. (g-i) Effect of different pulse intervals (0.25, 0.5, 1 μs) on EPSC, with constant pulse duration 0.5 μs, amplitude 3 V and number 40.

Excitatory postsynaptic currents (EPSC) play an important role in biological neuromorphology.^[4-5]^ When the applied stimulus stops, the EPSC slowly decays. Notably, when the applied stimulus is enhanced it causes the EPSC decay to become slower, similar to the learning memory process described above. The decay process of EPSC can be expressed by the following equation:$I\left( t \right)=I_{0}+Ae^{(-\frac{t}{\tau})}$，where τ represents the relaxation time constant, with larger values of τ representing slower decay, corresponding to a slower decrease in synaptic weights. **Figure S12** illustrates increasing the relaxation time by enlarging the pulse amplitude, lengthening the pulse duration, and decreasing the pulse spacing, which in turn slows down the forgetting of the EPSC, corresponding to enhancing the memory time of the HZO device.

**Figure S13** Pavlov's conditioned reflex experiment.

As shown in **Figure S13**, the pulse with an amplitude of 2 V, pulse width of 0.5 μs, and pulse interval of 0.5 μs is first designated as the food stimulus (non-conditioned stimulus); an amplitude of 4 V, pulse width of 1.5 μs, and pulse interval of 0.5 μs were designated as the bell sound (conditioned stimulus); an amplitude of 2 V with a pulse width of 0.5 μs and an amplitude of 4 V with a pulse width of 1.5 μs and pulse interval of 0.5 μs were designated as “food + bell sound” (conditioned stimulus + non-conditioned stimulus) for the training process. A threshold current of 4.64 μA has been established as the corresponding standard for evaluation.

First, ten unconditioned stimulus signals were applied, revealing that the device's current value remained below the threshold current, indicating that the dog did not secrete saliva in response to the ringing stimulus. Subsequently, after administering ten conditioned stimulus signals, the threshold current was exceeded, indicating that the dog produced saliva in response to the food stimulus as an unconditioned response. The device was then trained using both conditioned and unconditioned stimulus signals, thereby presenting food and bell stimuli simultaneously. During this training phase, the current consistently exceeded the threshold current, reflecting an unconditioned response to the conditioned stimulus signal. Following training, the response current elicited by the unconditioned stimulus signal alone surpassed the threshold current. Therefore, the Pavlovian classical conditioned response was successfully simulated using a ferroelectric memristor comprising Pd/HZO/LSMO/STO.

**Figure S14** (a) Device simulation of SRDP behavior under different frequency pulse stimulation. (3 V; 0.5 μs duration; pulse number: 10、20、10、5、10) (b) Device simulating SDDP behavior under different width pulse stimulation. (Amplitude 3V, interval 0.5μs constant, duration: 1 μs、2 μs、1 μs) (c) Device simulation of SADP behavior under different amplitude pulse stimulation. (Duration and interval: 0.5 μs; amplitude: 1 V、2 V、3 V、4 V、5 V)

**Figure S15** Schematic diagram of impulse application.

**Figure S16** Dot-line plots of the sampled values of the two features for 20 repetitions of the test with 16 different input pulse stimulus sequences applied to the HZO ferroelectric memristor.

**Figure S17** Loss functions of the three eigenvalue methods at 100 training cycles. (a) Loss function of the dual-feature method. (b) Loss function for eigenvalue SMP1 obtained by the first sampling point method. (c) Loss function for eigenvalue SMP2 obtained by the second sampling point method.

**Figure S18** Schematic of the 64 codons of the simulation program.

**Table 1** Comparison of parameters with other HfO_2_-based ferroelectric memristors.

| Device structure | Energy consumption | Multi states | Retention time | Pulse endurance | Ref. |
| --- | --- | --- | --- | --- | --- |
| HfO_2_/Al_2_O_3_/TiO_x_ | - | 4 | 10^4^ s | 10^6^ cycles | ^[6]^ |
| Pd/HfO_x_/NC/HfO_x_/TiN | - | 4 | 10^4^ s | 10^8^ cycles | ^[7]^ |
| Pt/HfO_2_/Pt | - | 2 | 10^4^ s | - | ^[8]^ |
| TiN/HfO_2_/HfO_2_:Al/Pt | - | 2 | 10^4^ s | 10^6^ cycles | ^[9]^ |
| Pd/Si:HfO_2_/LSMO/STO/Si | 32.65 fJ | 5 | 1.3×10^4^ s | 10^2^ cycles | ^[10]^ |
| (n-Si)/HfO_2_/WO_3_/Ag | - | 5 | 10^5^ s | 300 cycles | ^[11]^ |
| Au-HZO-Si | - | 5 | 7.2×10^3^ s | - | ^[12]^ |
| TiN/HZO/Pt | 1.8 pJ | - | - | 10^3^ cycles | ^[13]^ |
| TiN/HZO/TiN | 12.5 pJ | 4 | 4×10^3^ s | - | ^[14]^ |
| Pt/HfO_2_/ZrO_2-x_/HfO_2_/TiN | 14.5 nJ | 4 | 10^5^ s | 100 cycles | ^[15]^ |
| TaN/nc-HfO_2_/ITO | - | 2 | 10^4^ s | 250 cycles | ^[16]^ |
| Au/HZO/ITO | 113.4 fJ | - | - | - | ^[17]^ |
| W/HZO/SiO_2_/Si | 35 pJ | - | - | - | ^[18]^ |
| Pt/WSe_2_/HZO/TiN/Si | - | 2 | >10^4^ s | 2000 cycles | ^[19]^ |
| Ti/HZO/Pt | 60 pJ | 2 | >3×10^4^ s | 550 cycles | ^[20]^ |
| Pd/HZO/LSMO/STO | 121 fJ | 16 | >10^4^ s | 10^8^ cycles | This work |

**Table 2** Comparison of parameters with other ferroelectric memristors.

| Device structure | Energy consumption | Multi states | Retention time | Pulse endurance | Ref. |
| --- | --- | --- | --- | --- | --- |
| Au/P(VDF-TrFE)/NSTO | 100 fJ | 5 | 600 s | - | ^[21]^ |
| Au/BTO/LNO/Si | 0.365 pJ | - | - | - | ^[22]^ |
| MoS_2_/BST/SiO_2_/Si | 0.3 pJ | 2 | 3×10^3^ s | 34000 cycles | ^[23]^ |
| rGO/Ht-Mo_2_Ti_2_C_3_T_x_/Rgo | - | 2 | 5×10^3^ s | 10^3^ cycles | ^[24]^ |
| HSQ/MoS_2_/SiO_2_/P-Si | 3 pJ | - | - | - | ^[25]^ |
| Au/Co/BTO/LSMO/NdGaO_3_ | 100 fJ | - | - | 900 cycles | ^[26]^ |
| Pd/STO-MgO/LSMO/STO/Si | - | 12 | 10^4^ s | 10^9^ cycles | ^[27]^ |
| Ta/TaO_x_/Pt | - | - | - | 5×10^6^ cycles | ^[28]^ |
| Ag/SiO_2_/Pt | - | 2 | 10^4^ s | - | ^[29]^ |
| Au/SnSe/NSTO | 66 fJ | - | - | - | ^[30]^ |
| Ag/WS_2_/Pt | - | 2 | 1.1×10^3^ s | 100 cycles | ^[31]^ |
| LSMO/BTO/LSMO | 35 pJ | - | - | - | ^[32]^ |
| AlN/AlScN/AlN | 0.2 pJ | 4 | 10^4^ s | - | ^[33]^ |
| Au/Ti/AlScN/n-GaN | - | 2 | 10^3^ s | - | ^[34]^ |
| TiO_2_/BaTiO_4_/LNO/Si | 0.051 pJ | 6 | 10^4^ s | - | ^[35]^ |
| Pd/HZO/LSMO/STO | 121 fJ | 16 | >10^4^ s | 10^8^ cycles | This work |

**Table 3** Serial numbers from 1 to 64 stand in for the 64 codon recognition tasks.

| Serial number | Codon | Serial number | Codon | Serial number | Codon | Serial number | Codon |
| --- | --- | --- | --- | --- | --- | --- | --- |
| 1 | UUU | 17 | UCU | 33 | GCA | 49 | GAA |
| 2 | UUC | 18 | UCC | 34 | GCG | 50 | GAG |
| 3 | UUA | 19 | UCA | 35 | UAU | 51 | UGU |
| 4 | UUG | 20 | UCG | 36 | UAC | 52 | UGC |
| 5 | CUU | 21 | AGU | 37 | UAA | 53 | UGA |
| 6 | CUC | 22 | AGC | 38 | UAG | 54 | UGG |
| 7 | CUA | 23 | CCU | 39 | CAU | 55 | CGU |
| 8 | CUG | 24 | CCC | 40 | CAC | 56 | CGC |
| 9 | AUU | 25 | CCA | 41 | CAA | 57 | CGA |
| 10 | AUC | 26 | CCG | 42 | CAG | 58 | CGG |
| 11 | AUA | 27 | ACU | 43 | AAU | 59 | AGA |
| 12 | AUG | 28 | ACC | 44 | AAC | 60 | AGG |
| 13 | GUU | 29 | ACA | 45 | AAA | 61 | GGU |
| 14 | GUC | 30 | ACG | 46 | AAG | 62 | GGC |
| 15 | GUA | 31 | GCU | 47 | GAU | 63 | GGA |
| 16 | GUG | 32 | GCC | 48 | GAC | 64 | GGG |

**References:**

[1]	Z. Xia, X. Sun, Z. Wang, J. Meng, B. Jin, T. Wang, *Nano-Micro Letters* **2025**, 17, 217.

[2]	P. Gao, M. Duan, G. Yang, W. Zhang, C. Jia, *Nano Lett.* **2024**, 24, 10767.

[3]	N. Ilyas, J. Wang, C. Li, H. Fu, D. Li, X. Jiang, D. Gu, Y. Jiang, W. Li, *J. Mater. Sci. Technol.* **2022**, 97, 254.

[4]	H. So, S. Kim, S. Kim, *J. Alloys Compd.* **2024**, 1003, 175644.

[5]	Z. Shen, C. Zhao, L. Kang, Y. Sun, Y. Liu, I. Z. Mitrovic, L. Yang, E. G. Lim, Z. Zeng, C. Zhao, *Adv. Intell. Syst.* **2022**, 4, 2100236.

[6]	W. Banerjee, X. Zhang, Q. Luo, H. Lv, Q. Liu, S. Long, M. Liu, *Adv. Electron. Mater.* **2018**, 4, 1700561.

[7]	Q. Wu, W. Banerjee, J. Cao, Z. Ji, L. Li, M. Liu, *Appl. Phys. Lett.* **2018**, 113, 023105.

[8]	Y. Zhang, G.-Q. Mao, X. Zhao, Y. Li, M. Zhang, Z. Wu, W. Wu, H. Sun, Y. Guo, L. Wang, X. Zhang, Q. Liu, H. Lv, K.-H. Xue, G. Xu, X. Miao, S. Long, M. Liu, *Nat. Commun.* **2021**, 12, 7232.

[9]	Y.-L. Zhu, K.-H. Xue, X.-M. Cheng, C. Qiao, J.-H. Yuan, L.-H. Li, X.-S. Miao, *Appl. Surf. Sci.* **2021**, 550, 149274.

[10]	X. Yan, X. Jia, Y. Zhang, S. Shi, L. Wang, Y. Shao, Y. Sun, S. Sun, Z. Zhao, J. Zhao, J. Sun, Z. Guo, Z. Guan, Z. Zhang, X. Han, J. Chen, *Nano Energy* **2023**, 107, 108091.

[11]	Q. Liu, S. Gao, Y. Li, W. Yue, C. Zhang, H. Kan, G. Shen, *Adv. Mater. Technol.* **2022**, 8, 2201143.

[12]	T. Yu, F. He, J. Zhao, Z. Zhou, J. Chang, J. Chen, X. Yan, *Sci. China Mater.* **2020**, 64, 727.

[13]	L. Chen, T.-Y. Wang, Y.-W. Dai, M.-Y. Cha, H. Zhu, Q.-Q. Sun, S.-J. Ding, P. Zhou, L. Chua, D. W. Zhang, *Nanoscale* **2018**, 10, 15826.

[14]	S. Oh, T. Kim, M. Kwak, J. Song, J. Woo, S. Jeon, I. K. Yoo, H. Hwang, *IEEE Electron Device Lett.* **2017**, 38, 732.

[15]	T. Boynazarov, J. Lee, H. Lee, S. Lee, H. Chung, D. H. Ryu, H. Abbas, T. Choi, *J. Mater. Sci. Technol.* **2025**, 227, 164.

[16]	C. Mahata, M. Ismail, D. H. Kim, S. Kim, *J. Mater. Res. Technol.* **2022**, 21, 981.

[17]	M. Pei, Y. Zhu, S. Liu, H. Cui, Y. Li, Y. Yan, Y. Li, C. Wan, Q. Wan, *Adv. Mater.* **2023**, 35, 2305609.

[18]	J. Yu, Y. Li, W. Sun, W. Zhang, Z. Gao, D. Dong, Z. Yu,Y. Zhao, J. Lai, Q. Ding, Q. Luo, C. Dou, Q. Zuo, Y. Zhao, S. Chen, R. Zou, H. Chen, Q. Wang, H. Lv, X. Xu, D. Shang, M. Liu, IEEE Symposium onVLSITechnology (VLSI) 2021, T16-4.

[19]	D. Lee, S.-M. Kim, J.-C. Park, Y. Jung, S. Lee, B. H. Lee, S. Lee, *Appl. Surf. Sci.* **2025**, 679, 161216.

[20]	Q. Zhu, B. Jiang, J. Lan, Z. Hou, Y. Dong, Z. Wang, X. Feng, M. Shen, H. Yu, K. Chen, J. Li, L. Lin, F. Zhou, Y. Li, *Adv. Electron. Mater.* **2023**, 10, 2300508.

[21]	S. Majumdar, H. Tan, Q. H. Qin, S. van Dijken, *Adv. Electron. Mater.* **2019**, 5, 1800795.

[22]	D.-L. Li, X.-G. Tang, Q.-J. Sun, D.-P. Yang, Y.-P. Jiang, Q.-X. Liu, *Appl. Surf. Sci.* **2025**, 688, 162371.

[23]	C. Tan, H. Wu, M. Zhao, X. Jili, L. Yang, L. Gao, Z. Wang, *Adv. Funct. Mater.* **2024**, 34, 2405293.

[24]	K. Sattar, R. Tahir, S. A. Zahra, Z. Nie, J. Wang, H. Huang, S. Rizwan, *Carbon* **2025**, 237, 120149.

[25]	S. Subbulakshmi Radhakrishnan, A. Sebastian, A. Oberoi, S. Das, S. Das, *Nat. Commun.* **2021**, 12, 2143.

[26]	A. Chanthbouala, A. Crassous, V. Garcia, K. Bouzehouane, S. Fusil, X. Moya, J. Allibe, B. Dlubak, J. Grollier, S. Xavier, C. Deranlot, A. Moshar, R. Proksch, N. D. Mathur, M. Bibes, A. Barthélémy, *Nat. Nanotechnol.* **2011**, 7, 101.

[27]	Z. Guo, G. Liu, Y. Sun, Y. Zhang, J. Zhao, P. Liu, H. Wang, Z. Zhou, Z. Zhao, X. Jia, J. Sun, Y. Shao, X. Han, Z. Zhang, X. Yan, *ACS Nano* **2023**, 17, 21518.

[28]	D. S. Kuzmichev, Y. Y. Lebedinskii, C. S. Hwang, A. M. Markeev, *Phys. Status Solidi RRL* **2018**, 12, 1800429.

[29]	B. K. You, J. M. Kim, D. J. Joe, K. Yang, Y. Shin, Y. S. Jung, K. J. Lee, *ACS Nano* **2016**, 10, 9478.

[30]	H. Wang, W. Lu, S. Hou, B. Yu, Z. Zhou, Y. Xue, R. Guo, S. Wang, K. Zeng, X. Yan, *Nanoscale* **2020**, 12, 21913.

[31]	X. Yan, C. Qin, C. Lu, J. Zhao, R. Zhao, D. Ren, Z. Zhou, H. Wang, J. Wang, L. Zhang, X. Li, Y. Pei, G. Wang, Q. Zhao, K. Wang, Z. Xiao, H. Li, *ACS Appl. Mater. Interfaces* **2019**, 11, 48029.

[32]	W. Huang, W. Zhao, Z. Luo, Y. Yin, Y. Lin, C. Hou, B. Tian, C. G. Duan, X. G. Li, *Adv. Electron. Mater.* **2018**, 4, 1700560.

[33]	X. Dai, Q. Hua, C. Jiang, Y. Long, Z. Dong, Y. Shi, T. Huang, H. Li, H. Meng, Y. Yang, R. Wei, G. Shen, W. Hu, *Nano Energy* **2024**, 124, 109473.

[34]	M. Liu, S. Lu, Y. Jia, H. Zang, K. Jiang, X. Sun, D. Li, *IEEE Electron Device Lett.* **2024**, 45, 356.

[35]	D.-L. Li, X.-G. Tang, W.-M. Zhong, Q.-J. Sun, D.-P. Yang, Y.-P. Jiang, Q.-X. Liu, *ACS Sustainable Chem. Eng.* **2024**, 12, 13361.
